# Coronary Collateral Circulation in Patients of Coronary Ectasia with Significant Coronary Artery Disease

**DOI:** 10.1371/journal.pone.0087001

**Published:** 2014-01-27

**Authors:** Po-Chao Hsu, Ho-Ming Su, Hsiang-Chun Lee, Suh-Hang Juo, Tsung-Hsien Lin, Wen-Chol Voon, Wen-Ter Lai, Sheng-Hsiung Sheu

**Affiliations:** 1 Division of Cardiology, Department of Internal Medicine, Kaohsiung Medical University Hospital, Kaohsiung Medical University, Kaohsiung, Taiwan; 2 Graduate Institute of Medicine, College of Medicine, Kaohsiung Medical University, Kaohsiung, Taiwan; 3 Department of Medical Research, Kaohsiung Medical University Hospital, Kaohsiung Medical University, Kaohsiung, Taiwan; 4 Medical Genetics, Kaohsiung Medical University, Kaohsiung, Taiwan; 5 Center of Excellence for Environmental Medicine, Kaohsiung Medical University, Kaohsiung, Taiwan; 6 Faculty of Medicine, College of Medicine, Kaohsiung Medical University, Kaohsiung, Taiwan; 7 Department of Internal Medicine, Kaohsiung Municipal Hsiao-Kang Hospital, Kaohsiung, Taiwan; S.G.Battista Hospital, Italy

## Abstract

**Objectives:**

Patients with coronary ectasia (CE) usually have coexisting coronary stenosis resulting in myoischemia. Coronary collateral plays an important role in protecting myocardium from ischemia and reducing cardiovascular events. However, limited studies investigate the role of CE in coronary collaterals development.

**Methods:**

We evaluated 1020 consecutive patients undergoing coronary angiography and 552 patients with significant coronary artery disease (SCAD), defined as diameter stenosis more than 70%, were finally analyzed. CE is defined as the ectatic diameter 1.5 times larger than adjacent reference segment. Rentrop collateral score was used to classify patients into poor (grades 0 and 1) or good (grades 2 and 3) collateral group.

**Results:**

73 patients (13.2%) had CE lesions which were most located in the right coronary artery (53.4%). Patients with CE had a lower incidence of diabetes (43.8% vs 30.1%, p = 0.03), higher body mass index (25.4±3.5 vs 26.7±4.6, p = 0.027) and poorer coronary collateral (58.2% vs 71.2%, p = 0.040). Patients with poor collateral (n = 331) had a higher incidence of CE (15.7% vs 9.5%, p = 0.040) and fewer diseased vessels numbers (1.96±0.84 vs 2.48±0.69, p<0.001). Multivariate analysis showed diabetes (odd ratio (OR) 0.630, p = 0.026), CE (OR = 0.544, p = 0.048), and number of diseased vessels (OR = 2.488, p<0.001) were significant predictors of coronary collaterals development.

**Conclusion:**

The presence of CE was associated with poorer coronary collateral development in patients with SCAD.

## Introduction

Coronary ectasia (CE) is an uncommon disease and its incidence has been reported as between 0.3 and 5% in different studies despite some exception [1]–[5]. It is defined as the diameter of the ectatic segment being more than 1.5 times larger compared with an adjacent healthy reference segment [2]. Most cases of CE are considered as a variant of coronary artery disease (CAD) [6]. The pathogenesis of CE is not completely illustrated. However, it is likely to involve the destruction of the arterial media, increased wall stress, thinning of the arterial wall, and progressive dilatation of the coronary artery segment [7].

The development of coronary collaterals is an adaptive response to chronic myoischemia and serves as a conduit bridging the significantly stenotic coronary vessels [8]–[10]. Collateral circulation can hence protect and preserve myocardium from episodes of ischemia, enhance residual myocardial contractility, and reduce angina symptoms and cardiovascular events [11]–[13]. However, there is inter-individual difference of coronary collateral formation and the mechanisms for the different individual ability to develop collateral circulation are still unclear.

Because CE are usually associated with atherosclerosis and even obstructive CAD resulting coronary ischemia, whether the presence of good coronary collateral or not is a very important issue for the CE population [2], [6]. However, there were limited literatures discussing the coronary collateral formation in the CE population. Therefore we designed this study to investigate the role of CE in patients with obstructive CAD.

## Patients and Methods

### Study subjects

We evaluated 1020 patients scheduled for diagnostic coronary angiography from the Kaohsiung Medical University Hospital (KMUH) in Taiwan. Patients with coronary artery lumen diameter stenosis <70%, history of coronary artery bypass surgery (CABG), history of percutaneous coronary intervention (PCI), inadequate angiograms for CE evaluation were excluded. Finally 552 patients were recruited in our study. We collected patients' demographic and baseline information including sex, age, body mass index (BMI), duration of chest pain, history of diabetes, hypertension, hypercholesterolemia, and cigarette smoking.

### Ethics Statement

The research protocol was approved and registered by the Institutional Review Board of the Kaohsiung Medical University Hospital (KMUH-IRB). Informed consents were obtained in written form from patients and all clinical investigation was conducted according to the principles expressed in the Declaration of Helsinki. The patients gave consent for the publication of the clinical details.

### Coronary angiography

The coronary artery angiography films were reviewed by two experienced cardiologists blind to patients' clinical characteristics. A third reviewer blinded to the readings of the first two reviewers served as arbitrator of differences. Coronary angiography was performed by the femoral or radial approach with 6Fr diagnostic catheters. Images were recorded in multiple projections for left and right coronary arteries. Coronary artery stenosis was determined by quantitative coronary angiography. The presence of significant coronary artery disease (SCAD) is defined as coronary diameter stenosis more than 70%. CE is defined as the diameter of the ectatic segment being more than 1.5 times larger compared with an adjacent healthy reference segment [2]. The classification of CE developed by Markis et al. and based on the extent of ectatic involvement was used [14]. In decreasing order of severity, diffuse CE of two or three vessels was classified as Type I, diffuse disease in one vessel and localized disease in another vessel as Type II, diffuse CE of one vessel only as Type III and localized or segmental ectasia as Type IV. The recorded data also included the location, number of CAD and CE, percentage of stenosis of diseased vessels, the vessel to which the collaterals were connected, the grade of coronary collateral circulation, and the coronary artery disease severity scoring.

### Collateral scoring and pathways evaluation by coronary angiography

In subjects with more than one SCAD vessel, the vessel with the highest collateral grade was chosen for analysis. The collateral scoring system developed by Rentrop and Cohen was used [15]. Grades of collateral filling from the contralateral vessel were: 0 =  none; 1 =  filling of side branches of the artery to be dilated via collateral channels without visualization of the epicardial segment; 2 =  partial filling of the epicardial segment via collateral channels; 3 =  complete filling of the epicardial segment of the artery being dilated via collateral channels. In subjects with more than one collateral vessel supplying the distal aspect of the diseased artery, the highest collateral grade was recorded. Patients were then classified according to their collateral grades as either poor (grade 0 or grade 1 collateral) or good (grade 2 or grade 3 collateral). In addition, the size of the collateral connection (CC) diameter was assessed by 3 grades: CC grade 0, no continuous connection between donor and recipient artery; CC grade 1, continuous, threadlike connection, and CC grade 2, continuous, small side branch-like size of the collateral throughout its course [16]. In the case of coexisting collateral connections, the prominent one was defined as the principal. The anatomic pathways were categorized according to Levin's pathways and summarized in 4 categories: septal, intra-arterial (bridging), epicardial with proximal takeoff (atrial branches), and epicardial with distal takeoff [10], [17]. In the case of coexisting collateral pathways, the principal pathway was defined as the one that was the first to opacify the stenotic epicardial segments.

### Statistical analysis

All data were expressed as means ± standard deviation. Independent t test was used to compare continuous variables between the two groups. Chi-square test was used to compare categorical data. Subsequently, significantly correlated variables in the univariate analysis or relevant variables were further analyzed by binary logistic regression analysis to predict the collateral development (good vs. poor). All p values were two-sided with a significance level of p<.05. The Statistical Package for the Social Sciences 11.0 for Windows (SPSS Inc.,Chicago, IL) was used for statistical analysis.

## Results

### Clinical characteristics

Among the 1020 subjects initially evaluated, 468 patients were excluded for the following reasons: coronary artery lumen diameter stenosis <70%, history of CABG or PCI, or inadequate angiograms for collateral evaluation. The final study population was 552 subjects (443 male and 109 female; average age, 62.5±12.5 years old). Regarding numbers of diseased vessels, 148 patients (26.8%) were 1 vessel disease (VD), 163 patients (29.5%) were 2VD, and 241 patients (43.7%) were 3VD.

### Coronary ectasia and collaterals

There were 73 patients (13.2%) with CE with 24 patients (32.9%) 1VD, 26 (35.6%) 2VD, and 23 (31.5%) 3VD. Table 1 summarizes the angiographic characteristics of the patients with CE. 55 (75.3%) patients had CE involving one major vessel (right coronary artery: 39 patients, left anterior descending artery: 3 patient, left circumflex artery: 13 patients); 13 (17.8%) patients had CE involving two major vessel (right coronary artery and left anterior descending artery: 5 patients, right coronary artery and left circumflex artery: 7 patients, left anterior descending artery and left circumflex artery: 1 patients); 5 (6.8%) patients had CE involving all three vessels. For type of CE, 14 (19.2%) patients were type 1, 4 (5.5%) patients were type 2, 50 (68.5%) patients were type 3, and 5 (6.8%) patients were type 4. For coronary collateral grade and pathway evaluation, the 2 collaterals readers obtained a 96% agreement in the collateral classifications. Of the 73 patients with CE, the Rentrop coronary grade was distributed as follows: 40 (54.8%) patients with grade 0, 12 (16.4%) patients with grade 1, 13 (17.8%) patients with grade 2, and 8 (11%) patients with grade 3. The CC grade was distributed as follows: 49 (67.1%) patients with CC grade 0, 19 (26%) patients with CC grade 1, and 5 (6.8%) patients with CC grade 2. Furthermore, for the collateral pathways: the principal pathways was through septal connections in 57.6%, atrial-epicardial connections in 27.3%, bridging connections in 9.1%, and distal inter-arterial connections in 6.1%.

**Table 1:** Angiographic Characteristics of the Patients With Coronary Ectasia.

Ectasia(s) location	N (%)
Right coronary artery	39 (53.4%)
Left anterior descending artery	3 (4.1%)
Left circumflex artery	13 (17.8%)
Right coronary artery + left anterior descending artery	5 (6.8%)
Right coronary artery + left circumflex artery	7 (9.6%)
Left anterior descending artery + left circumflex artery	1 (1.4%)
All three vessels	5 (6.8%)
**Number of vessel(s) with coronary ectasia(s)**	
1	55 (75.3%)
2	13 (17.8%)
3	5 (6.8%)
**Type of coronary ectasia**	
Type 1	14 (19.2%)
Type 2	4 (5.5%)
Type 3	50 (68.5%)
Type 4	5 (6.8%)
**Rentrop collateral grade**	
Grade 0	40 (54.8%)
Grade 1	12 (16.4%)
Grade 2	13 (17.8%)
Grade 3	8 (11%)
**Collateral connection grades**	
CC0	49 (67.1%)
CC1	19 (26%)
CC2	5 (6.8%)
**Collateral pathway**	
Septal	19 (57.6%)
Atrial-epicardial	9 (27.3%)
Bridging	3 (9.1%)
Distal inter-arterial	2 (6.1%)

CC, collateral connection

Baseline characteristics in patients with and without CE were shown in the Table 2. The patients with CE (n = 73) had a lower incidence of DM (30.1% vs 43.8%, p = 0.030), higher BMI (26.7±4.6 vs 25.4±3.5, p = 0.027), and poor coronary collateral (71.2% vs 58.2%, p = 0.040). The multivariate regression analysis of coronary collateral formation (good vs poor collateral) in CE population with SCAD and found that only number of diseased vessels (OR = 2.358, 95% CI = 1.148–4.843, p = 0.02) was a significant independent predictor of coronary collaterals development (Table 3).

**Table 2:** Baseline Characteristics between Patients without and with Coronary Ectasia.

	Control	Ectasia	
Parameters N (%)	(n = 479)	(n = 73)	p Value
Sex (male)	379 (79.1)	64 (87.7)	0.113
Age (years)	62.8±12.1	60.3±14.8	0.174
DM	210 (43.8)	22 (30.1)	0.030
Hypertension	312 (65.1)	47 (64.4)	0.896
Smoking	280 (58.6)	50 (68.5)	0.124
Family history	16 (3.4)	2 (2.7)	1.000
Hypercholesterolemia (%)	264 (56.3)	36 (49.3)	0.311
CAD number			0.080
1-vessel disease	124 (25.9)	24 (32.9)	
2-vessel disease	137 (28.6)	26 (35.6)	
3-vessel disease	218 (45.5)	23 (31.5)	
BMI	25.4±3.5	26.7±4.6	0.027
Poor Collateral, n (%)	279 (58.2%)	52 (71.2%)	0.040

Data are presented as mean ± standard deviation or number (%); CAD, coronary artery disease; DM, diabetes mellitus; BMI, body mass index;

**Table 3:** Multivariate logistic regression analysis of collateral circulation in CE population (poor collateral group as reference group).

	OR	95% CI	p Value
Sex (male vs female)	-	-	0.896
Age	-	-	0.110
Hypertension	-	-	0.881
DM	-	-	0.178
Smoking	-	-	0.330
Hypercholesterolemia	-	-	0.291
BMI	-	-	0.652
Number of diseased vessels	2.358	1.148–4.843	0.020

BMI, body mass index; CE, coronary ectasia; DM, diabetes mellitus

### Coronary collaterals development in the whole population

Baseline characteristics in patients with poor and good collateral were shown in the Table 4, the patients with poor collateral (n = 331) had a higher incidence of CE (15.7% vs 9.5%, p = 0.040), and a fewer diseased vessels numbers (1.96±0.84 vs 2.48±0.69, p<0.001). Table 5 showed the multivariate analysis of coronary collateral formation (good vs poor collateral) in whole population with SCAD. We found diabetes (OR = 0.619, 95% CI = 0.410–0.934, p = 0.022), coronary ectasia (OR = 0.544, 95% CI = 0.297–0.996, p = 0.048), and number of diseased vessels (OR = 2.488, 95% CI = 1.917–3.228, p<0.001) were significant independent predictors of coronary collaterals development.

**Table 4:** Baseline Characteristics between good and poor collaterals.

	Poor	Good	
Parameters N (%)	(n = 331)	(n = 221)	p Value
Sex (male)	263 (79.5%)	180 (81.4%)	0.587
Age (years)	62.6±13.0	62.4±11.8	0.815
DM	143 (43.2%)	89 (40.3%)	0.538
Hypertension	214 (64.7%)	145 (65.6%)	0.856
Smoking	201 (60.7%)	129 (58.6%)	0.658
Family history	7 (2.1%)	11 (5.0%)	0.086
Hypercholesterolemia (%)	169 (52.3%)	131 (59.8%)	0.095
CAD number	1.96±0.84	2.48±0.69	<0.001
1-vessel disease	123 (37.2)	25 (11.3)	
2-vessel disease	97 (29.3)	66 (29.9)	
3-vessel disease	111 (33.5)	130 (58.8)	
BMI	25.5±3.74	25.7±3.6	0.553
Coronary ectasia, n (%)	52 (15.7%)	21 (9.5%)	0.040

Data are presented as mean ± standard deviation or number (%); CAD, coronary artery disease; DM, diabetes mellitus; BMI, body mass index

**Table 5:** Multivariate logistic regression analysis of collateral circulation in whole population (poor collateral group as reference group).

	OR	95% CI	p Value
Sex (male vs female)	-	-	0.395
Age	-	-	0.675
Hypertension	-	-	0.879
DM	0.619	0.410–0.934	0.022
Hypercholesterolemia	-	-	0.361
Smoking	-	-	0.270
BMI	-	-	0.318
Coronary ectasia	0.544	0.297–0.996	0.048
Number of diseased vessels	2.488	1.917–3.228	<0.001

BMI, body mass index; DM, diabetes mellitus

### Treatment strategies in CE patients

Among the 73 patients with CE, 58 patients (79.5%) received PCI, 8 patients (11%) received CABG, and 7 patients (9.6%) only received medical treatment for the coronary lesions. There was no significant association between treatment strategies and presence of good or poor coronary collaterals (p = 0.284).

## Discussion

There were three major findings in the present study. First, most CE lesion were located in the right coronary artery. Second, CE patients with significant coronary artery disease have poor coronary collateral development compared with non-CE patients. Third, CE is a significantly independent predictor of poor coronary collaterals in patients with SCAD.

### The association between CE and coronary collaterals

CE is a variant of coronary artery abnormality [6]. It may be congenital or acquired. Acquired causes include atherosclerosis, Kawasaki disease, various inflammatory and infectious diseases, and so on [2]–[6]. However, most cases of CE are associated with atherosclerosis and had coexistence of obstructive CAD [2], [6]. It was considered to be a variant of CAD and is associated with the similar risk as the patients with CAD [2], [14], [18]–[19]. The clinical presentation and the long-term cardiac complications are associated with the severity of coexisting CAD [20]. Even in patients with isolated CE without coronary stenosis, there is still higher incidence of adverse events in this population compared to people with normal coronary arteries. The pathogenesis of CE is not completely understood, however, it is likely to involve the destruction of the arterial media, increased wall stress, thinning of the arterial wall, and progressive dilatation of the coronary artery segment^7^. The presence of ectatic segments produces sluggish blood flow, with exercise-induced angina and myocardial infarction, regardless of the severity of coexisting obstructive CAD [14. 18–19]. The possible causes of higher adverse events might be related to the repeated dissemination of microemboli to distal segments, or thrombotic occlusion of the dilated vessel. In addition, slow blood flow in the ectatic coronary arteries might be another cause which predisposes to the occurrence of AMI. Disturbances in blood flow filling and washout in the ectatic vessels were due to inappropriate coronary dilatation and were clearly associated with the severity of CE [21]. The turbulent and stagnant blood flow could induce endothelial damage, increase wall stress, and even cause extensive thrombosis. In past literature, all three coronary arteries can be affected by CE, but most patients had single-vessel involvement [22.23]. Furthermore, In CE patients with coexistent CAD, the right coronary artery is the most frequently involved. These findings were also similar with our current study.

The development of coronary collaterals can reduce angina and infarct size, preserve left ventricular ejection fraction, decrease aneurysmal dilatation, and provide a survival benefit in patients with SCAD [11]–[13]. However, there are limited studies discussing about the coronary collateral formation in the CE population and most of the studies were focused on the young patients with Kawasaki disease [24]–[25]. In children with Kawasaki disease, Onouchi Z et al. reported that coronary collaterals did not develop in the presence of localized stenosis regardless of the occurrence of myocardial ischemia, but total occluded vessels had collateral development regardless of the presence of myocardial infarction [24]. Tatara K et al. also indicated that collateral circulation cannot be seen angiographically unless there is total occlusion and the presence of collateral circulation cannot provide protection against stress-induced myocardial ischemia [25]. These findings all suggest that relative poor collateral formation in the patients with etiology of Kawasaki disease. Despite of the etiologies different from previous studies, our study showed that CE is significantly associated with poor coronary collateral formation both in univariate and multivariate analysis. Although CE is reported to be associated with increased plasma levels of inflammatory markers, cytokines, and oxidative stress, the detailed mechanism of poor coronary collateral development is not well understood nowadays and may need further investigation in the future [26]–[28].

In addition, number of diseased vessels was not only a significant predictor of coronary collateral formation in the patients with SCAD, but also the only significant predictor in the CE population with SCAD. It is well known that coronary collateral formation is mainly dependent on the CAD severity [29]–[31]. Patients with good coronary collaterals appear to have a more extensive CAD. In previous studies, number of diseased vessels is a significant predictor of good collateral formation [32], [33]. Hence, extent of coronary atherosclerosis significantly plays an important role in coronary collateral formation.

### Limitations of the present study

First, the collateral formation was assessed by coronary angiography in this study. Measuring collateral flow index by intravascular Doppler guidewire may provide a more objective physiological measurement of collateral grade. However, the invasiveness of intravascular ultrasound limits its use in large-scale studies. Second, since this was only a clinical association study, potential mechanisms were not fully elucidated and long term follow-up of clinical outcome data will be needed to see whether CE patients with poorer coronary collaterals have higher incidence of cardiovascular adverse events in the future.

### Conclusions

To our best, our study is the first study to show the poor coronary collateral development in the adult patients with CE and SCAD. Our data might partially explain why patients with CE have higher incidence of cardiovascular adverse events.
